# A Novel Six Autophagy-Related Genes Signature Associated With Outcomes and Immune Microenvironment in Lower-Grade Glioma

**DOI:** 10.3389/fgene.2021.698284

**Published:** 2021-10-13

**Authors:** Tao Lin, Hao Cheng, Da Liu, Lei Wen, Junlin Kang, Longwen Xu, Changguo Shan, Zhijie Chen, Hainan Li, Mingyao Lai, Zhaoming Zhou, Weiping Hong, Qingjun Hu, Shaoqun Li, Cheng Zhou, Jiwu Geng, Xin Jin

**Affiliations:** ^1^ Department of Neurosurgery, Guangdong Sanjiu Brain Hospital, Guangzhou, China; ^2^ Department of Nasopharyngeal Carcinoma, The First People’s Hospital of Chenzhou, Southern Medical University, Chenzhou, China; ^3^ Department of Oncology, Guangdong Sanjiu Brain Hospital, Guangzhou, China; ^4^ Department of Neurosurgery, Lanzhou University First Hospital, Lanzhou, China; ^5^ Department of Medical Oncology, The First Affiliated Hospital of Xi’an Jiaotong University, Xi’an, China; ^6^ Department of Pathology, Guangdong Sanjiu Brain Hospital, Guangzhou, China; ^7^ Department of Radiation Oncology, Nanfang Hospital, Southern Medical University, Guangzhou, China; ^8^ Guangdong Key Laboratory of Occupational Disease Prevention and Treatment/Guangdong Province Hospital for Occupational Disease Prevention and Treatment, Guangzhou, China

**Keywords:** lower-grade glioma, gene signature, autophagy, immune microenvironment, immunotherapy

## Abstract

Since autophagy and the immune microenvironment are deeply involved in the tumor development and progression of Lower-grade gliomas (LGG), our study aimed to construct an autophagy-related risk model for prognosis prediction and investigate the relationship between the immune microenvironment and risk signature in LGG. Therefore, we identified six autophagy-related genes (BAG1, PTK6, EEF2, PEA15, ITGA6, and MAP1LC3C) to build in the training cohort (*n* = 305 patients) and verify the prognostic model in the validation cohort (*n* = 128) and the whole cohort (*n* = 433), based on the data from The Cancer Genome Atlas (TCGA). The six-gene risk signature could divide LGG patients into high- and low-risk groups with distinct overall survival in multiple cohorts (all *p* < 0.001). The prognostic effect was assessed by area under the time-dependent ROC (t-ROC) analysis in the training, validation, and whole cohorts, in which the AUC value at the survival time of 5 years was 0.837, 0.755, and 0.803, respectively. Cox regression analysis demonstrated that the risk model was an independent risk predictor of OS (HR > 1, *p* < 0.05). A nomogram including the traditional clinical parameters and risk signature was constructed, and t-ROC, C-index, and calibration curves confirmed its robust predictive capacity. KM analysis revealed a significant difference in the subgroup analyses’ survival. Functional enrichment analysis revealed that these autophagy-related signatures were mainly involved in the phagosome and immune-related pathways. Besides, we also found significant differences in immune cell infiltration and immunotherapy targets between risk groups. In conclusion, we built a powerful predictive signature and explored immune components (including immune cells and emerging immunotherapy targets) in LGG.

## Introduction

Diffuse low-grade and intermediate-grade gliomas including WHO grades II and III, hereafter called lower-grade gliomas (LGG) (Cancer Genome Atlas Research et al., 2015). Lower-grade gliomas (LGG) constitute about 15 percent of all primary brain tumors that originate from glial cells, showing great heterogeneity in clinical outcomes (Ostrom et al., 2013; Zeng et al., 2018). So far, maximum surgery, subsequent-radiotherapy, and chemotherapy have been the standard treatment modalities for LGG (Soffietti et al., 2010). Although numerous efforts to prolong LGG patient survival, more than half of them develop and progress to treatment-resistant and aggressive high-grade glioma in the future (Claus et al., 2015). Hence, it is urgent to search for novel prognostic biomarkers and therapeutic targets of LGG. Several genetic biomarkers were incorporated into the 2016 WHO classification, including chromosome arms 1p and 19q codeletion, isocitrate dehydrogenase (IDH) mutation, and O-6-methylguanine-DNA methyltransferase (MGMT) methylation, to illuminate the histological characteristics and guide the therapeutic approach (Hartmann et al., 2010; Wick et al., 2013; Hainfellner et al., 2014; Louis et al., 2016). Although these widely utilized biomarkers in LGG have recently been discovered, the novel predictors of clinical outcomes or therapeutic targets for LGG are not fully unraveled.

Autophagy is a highly conserved lysosomal degradation process that is crucial for homeostasis, differentiation, development, and survival (Rabinowitz and White, 2010) and has been found involved in diverse pathologies, including cancer (Kondo et al., 2005). By self-degradation of damaged proteins and intracellular components, autophagy can suppress tumor initiation, thereby mitigating cell injury and suppressing chromosomal instability (Mathew et al., 2007; White et al., 2010). But, autophagy can also facilitate cancer proliferation by supplying nutritional substance in the context of hypoxic and innutritious surroundings (Guo et al., 2011). Mostly, autophagy is believed to impede cancer initiation and promote tumor progression (Trejo-Solis et al., 2018). In addition, autophagy can alter the tumor or stroma cell immunogenicity within the tumor microenvironment (TME) and the development of antitumor immunity through intertwining with pattern recognition receptor (PRR), cell death pathways, and inflammatory (Gerada and Ryan, 2020). Nevertheless, few studies have reported the impact on prognosis and the correlation with immune cells of autophagy in LGG.

In the study, we established a powerful prognostic signature based on six autophagy-related genes, and then a nomogram was built with the signature and traditional clinical parameters, to predict clinical outcomes and assist clinical procedures. Moreover, the association of autophagy-related genes signature with immune cells and emerging immune targets and was further analyzed.

## Materials and Methods

### Data Collection and Processing

The level 3 RNA-seq expression profiles and corresponding clinicopathologic data including age, gender, grade, IDH mutation status, chemotherapy, radiotherapy of LGG patients were obtained from TCGA Lower Grade Glioma (LGG) of UCSC Xena (https://xenabrowser.net/). All patients were diagnosed with LGG, who were followed for more than 90 days and have complete clinical information. Overall, 433 patients of the LGG whole cohort met the screening rules. The patients were randomly separated into a training cohort (*n* = 305) and a validation cohort (*n* = 128) at a ratio of 7:3. mRNA Expression profiles used in normal brain tissues were downloaded from the Genome Tissue Expression (GTEx, https://gtexportal.org/home/datasets) (Consortium, 2015). To normalize expression data and eliminate the batch effects, the “sva” R package was used.

### Selection and Functional Enrichment of Autophagy-Related Genes

The “limma” R package was employed to select differentially expressed genes (DEGs) by comparing TCGA-LGG tissues and GTEX-brain normal tissues, with the included criteria (Adj. *p* < 0.05 and |LogFC| > 1) (Ritchie et al., 2015). A volcano plot was used to visualize the DEGs. The 232 autophagy-related genes (ARGs) were extracted from the Human Autophagy Database (HADb, http://www.autophagy.lu/) (Moussay et al., 2011). The intersection of the DEGs and ARGs was selected as the significant differentially expressed autophagy-related genes (DE-ARGs) for further assessment and was then showed in Venn diagrams.

In the whole set, LGG patients were separated into two risk groups, low- and high-risk groups, according to the optimal risk cutoff obtained from the training set. To probe underlying functions of DE-ARGs and risk model, the biological process of GO and KEGG pathways analysis was performed and GESA was conducted to identify the critical altered signaling pathways between high- and low-risk groups, by the aid of the “clusterProfiler” package in R 3.6.3 (Yu et al., 2012). The “c2.cp.kegg.v7.0.symbols.gmt” KEGG gene set was adopted as reference. The nominal *p*-value (NOM-*P*) for gene sets <0.05, the absolute normalized enrichment score (|NES|) > 1.8 and the false discovery rate (FDR) <0.05 were confirmed as threshold.

### Construction and Validation of the Risk Model Based on Autophagy-Related Genes

Performing univariate Cox regression analysis in the “survival” R package, 13 of 53 DE-ARGs in the training cohort was identified with prognosis significance (all *p* < 0.05) (Linden and Yarnold, 2017). The least absolute shrinkage and selection operator (LASSO) (Friedman et al., 2010) analysis was utilized to establish the risk model. The prognostic risk score model according to a combination of LASSO coefficient and the corresponding normalized expression level was built in the following equation: risk score = sum (the normalized expression level of each gene × corresponding LASSO coefficient). Subsequently, a risk score was computed for each patient. All patients were stratified into the low-risk and high-risk groups based on the optimum cutoff of risk score (risk score = −7.009) counted by ROC curve using the “survminer” package in R (Supplementary Figure S1). Next, a KM plot based on log-rank test was applied to measure the survival difference between patients with high- and low-risk groups. The prognostic capacity of the ARG-based signature was investigated by using Harrell’s concordance index (C-index), time-dependent receiver operating characteristic (ROC) curve, and Principal component analysis (PCA) with the R packages “survcomp,” “survivalROC,” and “ scatterplot3d” (Harrell et al., 1996; Mächler and Ligges, 2003; Alba et al., 2017). Then, the prognostic effect of the signature established by the training set was verified in the validation cohort and the whole cohort using some similar methods.

Moreover, to evaluated whether the predictive capacity of the prognostic risk model could be independent of other clinic factors (including age, gender, WHO grade, radiotherapy, chemotherapy, and IDH status) for patients with LGG, univariate Cox regression and multivariate Cox regression analyses were applied in the TCGA training cohort, the validation cohort, and the whole cohort. Next, by using “rms,” “foreign,” and “survival” R packages, we established a nomogram comprising of traditional clinical factors and risk score based on the multivariate Cox regression analysis. The prognostic effect of the prognostic nomogram was examined by Harrell’s concordance index (C-index), time-dependent ROC curve, and calibration plots of the nomogram for 3 and 5 years OS plotted to assess the coincidence of actual observed rates with the predicted survival probability. Time-dependent ROC analyses were performed by the “timeROC” R package.

### Associations Between Immune Components and Autophagy-Related Genes Signature

To identify the potential association between the signature and immune components, both emerging immune targets and tumor-infiltrating immune cells were included. The list of potential immunotherapy targets involved in innate and adaptive immune processes was extracted from a recent review (Burugu et al., 2018). We Compared the target gene expression between different risk groups. CIBERSORT algorithm, a novel deconvolution algorithm, uses 547 reference gene expression values for estimating enrichment of different immunocyte subpopulations (Newman et al., 2015). Our study applied the CIBERSORT algorithm to examine the abundance of 22 infiltrating immune cells in the high-risk and low-risk group in the whole cohort. Utilizing the Monte-Carlo sampling, the deconvolution *p*-values of samples were computed to offer reliability in the assessment. Patients with *p* < 0.05 were considered to be high reliability of the inferred cell composition. Therefore, samples with a *p* value of <0.05 were retained for subsequent analysis. The expression profiles of TCGA-LGG patients were put on the CIBERSORT web tool (http://cibersort.stanford.edu/) for analysis with the default signature matrix at 1,000 permutations.

### Statistical Analysis

All data analyses were done on software R (version 3.6.3). The student’s t-test and chi-square test were used to determine that whether there is a difference in clinical parameters between the training cohort and validation cohort and to evaluate the association between clinical characteristics and the risk score. Kaplan–Meier survival analysis was used to compare the prognosis between risk groups. The significantly independent prognostic factors in LGG were identified using univariate and multivariate Cox regression. The predictive capacity of the signature and other clinical parameters was determined by ROC curves. A nomogram was constructed with the “rms” package in R, by using multivariate Cox analysis. The C-index and calibration plot with the bootstrap method were performed to evaluate the predictive power of the nomogram. A *p* value <0.05 is considered statistically significant.

## Results

### Identification of Differentially Expressed Autophagy-Related Genes and Enrichment Analysis

**FIGURE 1:**
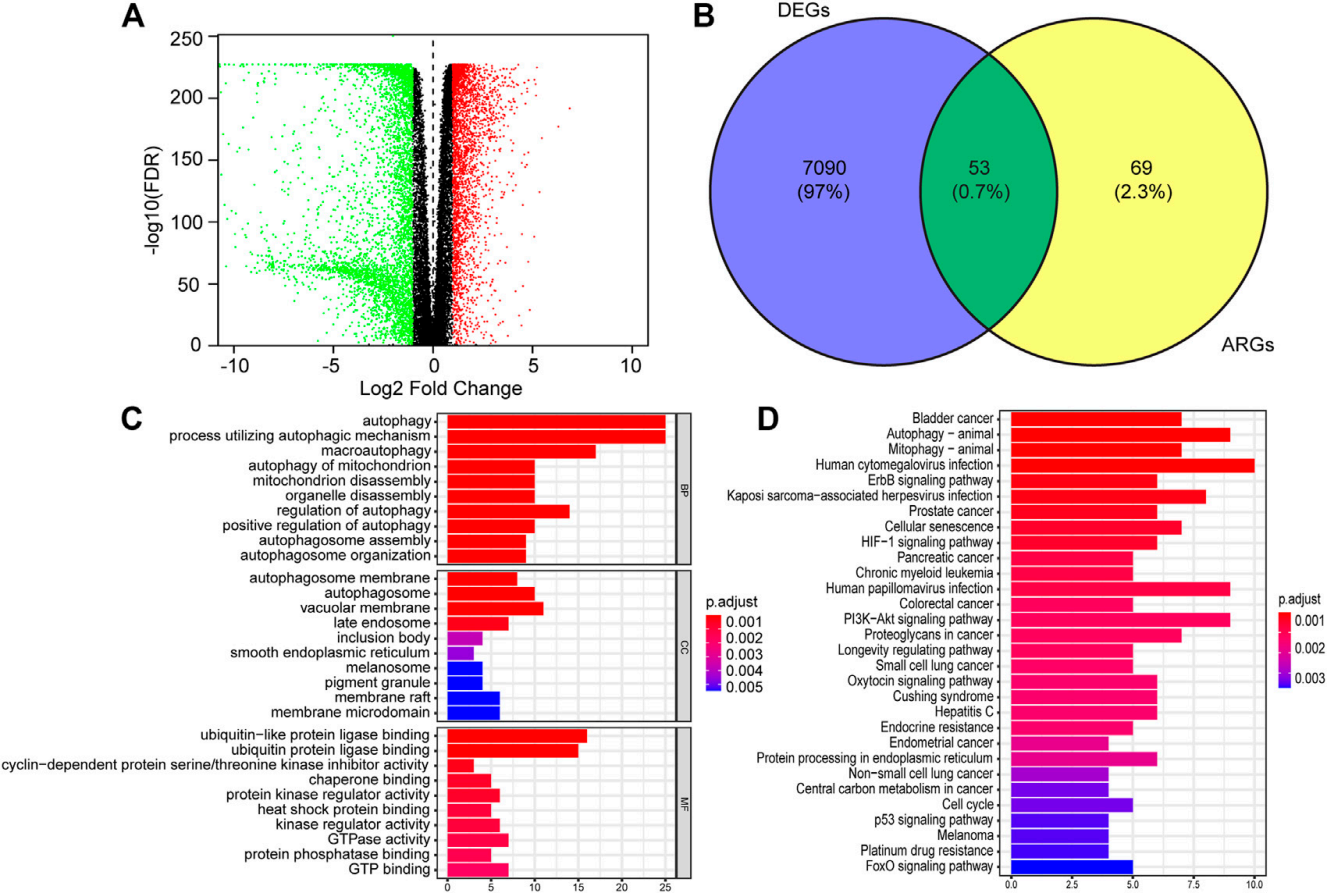
Identification of differentially expressed autophagy-related genes (DE-ATGs) in low grade glioma (LGG) and enrichment analysis. **(A)** Volcano plot of DEGs in 529 tumor tissues of The Cancer Genome Atlas (TCGA) dataset and 1,035 normal samples from The Genotype-Tissue Expression (GTEx). The vertical axis indicates the–log [adjusted *p* value (adj. *p* value)], and the horizontal axis indicates the log2 [fold change (FC)]. The red dots represent upregulated genes, and the green dots represent downregulated genes (adj. *p* value <0.01 and |log2(FC)| > 1). **(B)** Venn diagram showing the 53 DE-ARGs (the intersection of the DEGs and ARGs). **(C)** Biological processes (BP), Cellular components (CC) and Molecular functions (MF) enriched in the DE-ARGs. **(D)** Kyoto Encyclopedia of Genes and Genomes (KEGG) pathways enriched in the DE-ARGs.

RNA-seq expression data and clinical information of 529 lower-grade glioma tissue samples were obtained from TCGA, and 1,035 non-tumor samples were selected from GTEX. Of those patients, a total of 433 LGG patients who were followed for more than 3 months and had complete clinical data were analyzed in the study. After analyzing the TCGA-LGG expression data using limma, 7,143 DEGs were found between LGG and normal tissues and showed in the volcano plots (Figure 1A). Venn diagrams revealed that the intersection of fifty-three significant DE-ARGs were used for further analysis (Figure 1B).

Next, we performed functional enrichment analysis to identify risk pathways and biological functions associated with the DE-ARGs. Go enrichment analysis revealed that the biological process of the DE-ARGs were significantly enriched in terms of autophagy-related processes; the cellular component of the DE-ARGs were significantly enriched in the terms autophagosome membrane, autophagosome and vacuolar membrane and the molecular function of the DE-ARGs were significantly enriched in the terms ubiquitin and ubiquitin−like protein ligase binding, and cyclin−dependent protein serine/threonine kinase inhibitor activity (Figure 1C). In addition, KEGG enrichment analysis showed that the DE-ARGs were mainly involved in cancer-related pathways, Autophagy−animal and Mitophagy–animal (Figure 1D).

### Establishment of an Autophagy-Related Model for Survival Prediction in the The Cancer Genome Atlas Lower-Qrade Qliomas Training Cohort

According to the screening conditions, we randomly separated 433 patients in TCGA-LGG into a training dataset (*n* = 305) and a validation dataset (*n* = 128), using the “caret” package. The chi-square test demonstrated no significant difference in basic clinical factors between the two datasets (Table 1). Moreover, the clinicopathological parameters of LGG patients based on risk signature constructed below was also examined (Supplementary Table S2). After univariate Cox regression analysis, 13 significantly prognosis-associated genes were identified in the training cohort of 305 LGG patients. These significant genes entered into LASSO COX regression analyses, and the regression coefficient was determined. As a result, the six most important genes were identified as BAG Cochaperone 1 (BAG1), Protein Tyrosine Kinase 6 (PTK6), Eukaryotic Translation Elongation Factor 2 (EEF2), Proliferation and Apoptosis Adaptor Protein 15 (PEA15), Integrin Subunit Alpha 6 (ITGA6), and Microtubule Associated Protein 1 Light Chain 3 Gamma 5 (MAP1LC3C). An autophagy-related LGG risk score was constructed through a linear combination of the expression values of the six autophagy-related genes adjusted by the LASSO regression coefficient. The risk score = −0.7733 × expression level of BAG1-0.2010 × expression level of PTK6-0.1621 × expression level of EEF2-0.4639 × expression level of PEA15 + 0.0565 × expression level of ITGA6 + 0.3223 × expression level of MAP1LC3C. The risk score for each patient was calculated according to this equation (Table 2).

**TABLE 1:** Demographics and clinicopathological data of 433 LGG patients from the TCGA database.

Clinical variables	Total set number (%)	Training set number (%)	Validating set number (%)	*p* Value
Age at diagnosis				
<40	237 (54.73)	159 (52.13)	78 (60.94)	0.1155
≥40	196 (45.27)	146 (47.87)	50 (39.06)	
Gender				
FEMALE	197 (45.5)	135 (44.26)	62 (48.44)	0.4899
MALE	236 (54.5)	170 (55.74)	66 (51.56)	
Grade				
G2	206 (47.58)	141 (46.23)	65 (50.78)	0.4473
G3	227 (52.42)	164 (53.77)	63 (49.22)	
Radiotherapy				
NO	159 (36.72)	113 (37.05)	46 (35.94)	0.9126
YES	274 (63.28)	192 (62.95)	82 (64.06)	
Chemotherapy				
NO	182 (42.03)	134 (43.93)	48 (37.5)	0.258
YES	251 (57.97)	171 (56.07)	80 (62.5)	
IDH status				
Mutation	353 (81.52)	246 (80.66)	107 (83.59)	0.5598
Wild	80 (18.48)	59 (19.34)	21 (16.41)	

**TABLE 2:** Six survival-related autophagy-related gene in the signature associated with overall survival in the TCGA-training set.

ID	uniCox regression				LASSO
	HR	Low 95% CI	High 95% CI	*p* value	Coefficient
BAG1	0.060069	0.023310698	0.154791644	5.78E-09	−0.773344244
PTK6	0.175985	0.084795649	0.365238271	3.11E-06	−0.200973447
EEF2	0.362671	0.241460173	0.544727587	1.02E-06	−0.162054564
PEA15	0.335272	0.238622505	0.471066233	3.00E-10	−0.463926374
ITGA6	3.111707	1.844873426	5.248445579	2.08E-05	0.056506153
MAP1LC3C	2.274053	1.794350072	2.881999107	1.07E-11	0.322322447

**FIGURE 2:**
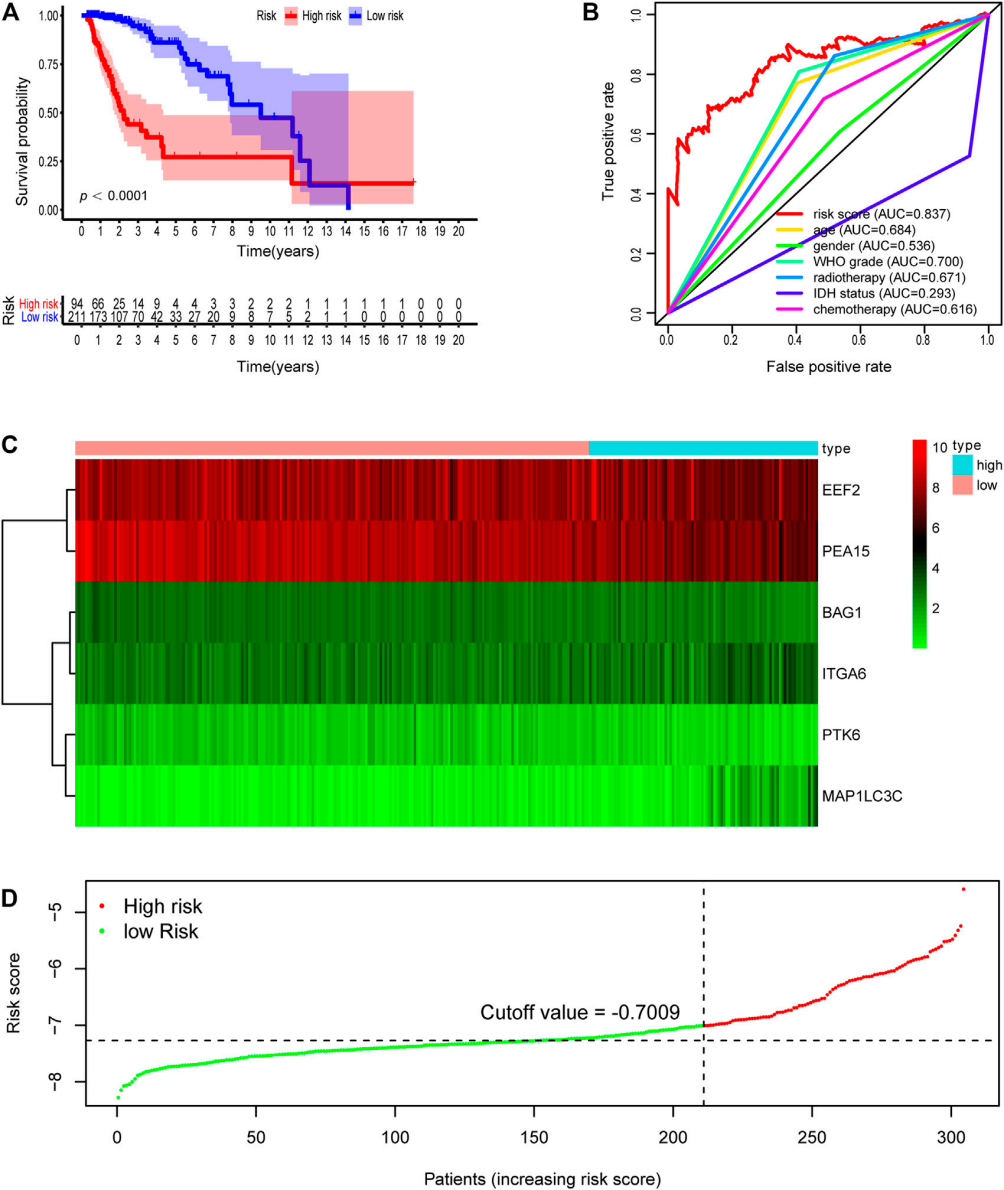
Development of risk score based on the six autophagy-related gene signature of patients with TCGA-LGG training set. **(A)** Kaplan-Meier plot for overall survival (OS) based on risk score of the six gene based signature of patients with TCGA-LGG training cohort. **(B)** ROC curve for 5 years OS in training cohort. **(C)** Heatmap of the six autophagy-related gene expression in the training cohort. **(D)** Risk plot of each point sorted based on risk score. The black dotted line is the optimal cutoff (−7.009) classifying patients into low risk and high risk groups.

Subsequently, we computed the risk score for each LGG patient in the training cohort. The cutoff risk score (−7.009) was counted using the “survminer” package in the TCGA-LGG training cohort. All LGG patients were then separated into low- (risk score < −7.009) and high-risk (risk score ≥ −7.009) groups (Figure 2D). Kaplan–Meier survival analysis showed that patients in high-risk group were associated with a relatively poor OS as than those in the low-risk group (log-rank *p* = 1.554e-15, Figure 2A). The heatmap showed that six prognostic expression profiles between two risk groups (Figure 2C). Besides, multivariate Cox regression analysis demonstrated that the risk score could independently predict OS after adjusting for various clinicopathologic parameters in the training cohort (Table 3). ROC analysis of 5 years overall survival was applied to examine the predictive capacity of the six-gene prognostic risk model. Moreover, the 5 years AUC of risk model was 0.837, which was markedly higher than that of age (AUC = 0.684), gender (AUC = 0.538), WHO grade (AUC = 0.700), radiotherapy (AUC = 0.671), IDH status (AUC = 0.293), and chemotherapy (AUC = 0.616), indicating that it has a more robust prediction of clinical outcome than the other clinical parameters (Figure 2B).

**TABLE 3:** Univariate and multivariate Cox regression analysis in TCGA-LGG each cohorts.

Variables	Univariate analysis		Multivariate analysis	
	HR (95% CI)	*p* Value	HR (95% CI)	*p* Value
Training set (*n* = 305)				
Age (<40/≥40)	3.908 (2.214–6.898)	2.5965E-06	2.750 (1.448–5.222)	0.00198857
Gender (female/male)	1.250 (0.765–2.042)	0.372437476	1.974 (1.160–3.360)	0.01221692
Grade (G2/G3)	3.459 (1.972–6.066)	1.49742E-05	1.774 (0.919–3.422)	0.08750485
Radiotherapy (no/yes)	3.045 (1.583–5.854)	0.000844437	1.507 (0.710–1.131)	0.28553912
IDH status (wild/mutation)	0.143 (0.088–0.234)	9.56649E-15	0.465 (0.191–1.131)	0.09129914
Chemotherapy (no/yes)	1.628 (0.977–2.712)	0.061242815	0.820 (0.470–1.432)	0.4854889
Risk score (low/high)	4.645 (3.353–6.435)	2.59641E-20	2.493 (1.336–4.651)	0.0041002
Validation set (*n* = 128)				
Age (<40/≥40)	3.425 (1.427–8.220)	0.005861356	3.319 (1.097–10.05)	0.03372453
Gender (female/male)	0.794 (0.377–1.673)	0.544405373	1.059 (0.461–2.431)	0.89283856
Grade (G2/G3)	3.572 (1.572–8.117)	0.002367324	2.376 (0.817–6.911)	0.11217943
Radiotherapy (no/yes)	1.775 (0.761–4.140)	0.183865679	1.921 (0.669–5.517)	0.22532202
IDH status (wild/mutation)	0.116 (0.047–0.288)	3.63036E-06	0.776 (0.139–4.338)	0.77242088
Chemotherapy (no/yes)	0.865 (0.421–1.778)	0.693438967	0.218 (0.086–0.553)	0.00131428
Risk score (low/high)	4.334 (2.546–7.381)	6.64509E-08	3.583 (1.151–11.16)	0.02762528
Whole set (*n* = 433)				
Age (<40/≥40)	3.541 (2.243–5.590)	5.70336E-08	2.918 (1.717–4.957)	7.4951E-05
Gender (female/male)	1.060 (0.713–1.576)	0.772843899	1.472 (0.964–2.248)	0.07342518
Grade (G2/G3)	3.307 (2.213–5.151)	1.22602E-07	1.880 (1.115–3.170)	0.01789178
Radiotherapy (no/yes)	2.535 (1.516–4.239)	0.000389848	1.484 (0.818–2.692)	0.19380046
IDH status (wild/mutation)	0.147 (0.098–0.222)	5.54714E-20	0.560 (0.272–1.154)	0.11614564
Chemotherapy (no/yes)	1.333 (0.881–2.016)	0.173180772	0.623 (0.394–0.985)	0.04287472
Risk score (low/high)	4.593 (3.487–6.050)	2.04139E-27	2.714 (1.644–4.482)	9.5588E-05

### Testing the Signature in the Validation Cohort and the Whole Cohort

**FIGURE 3:**
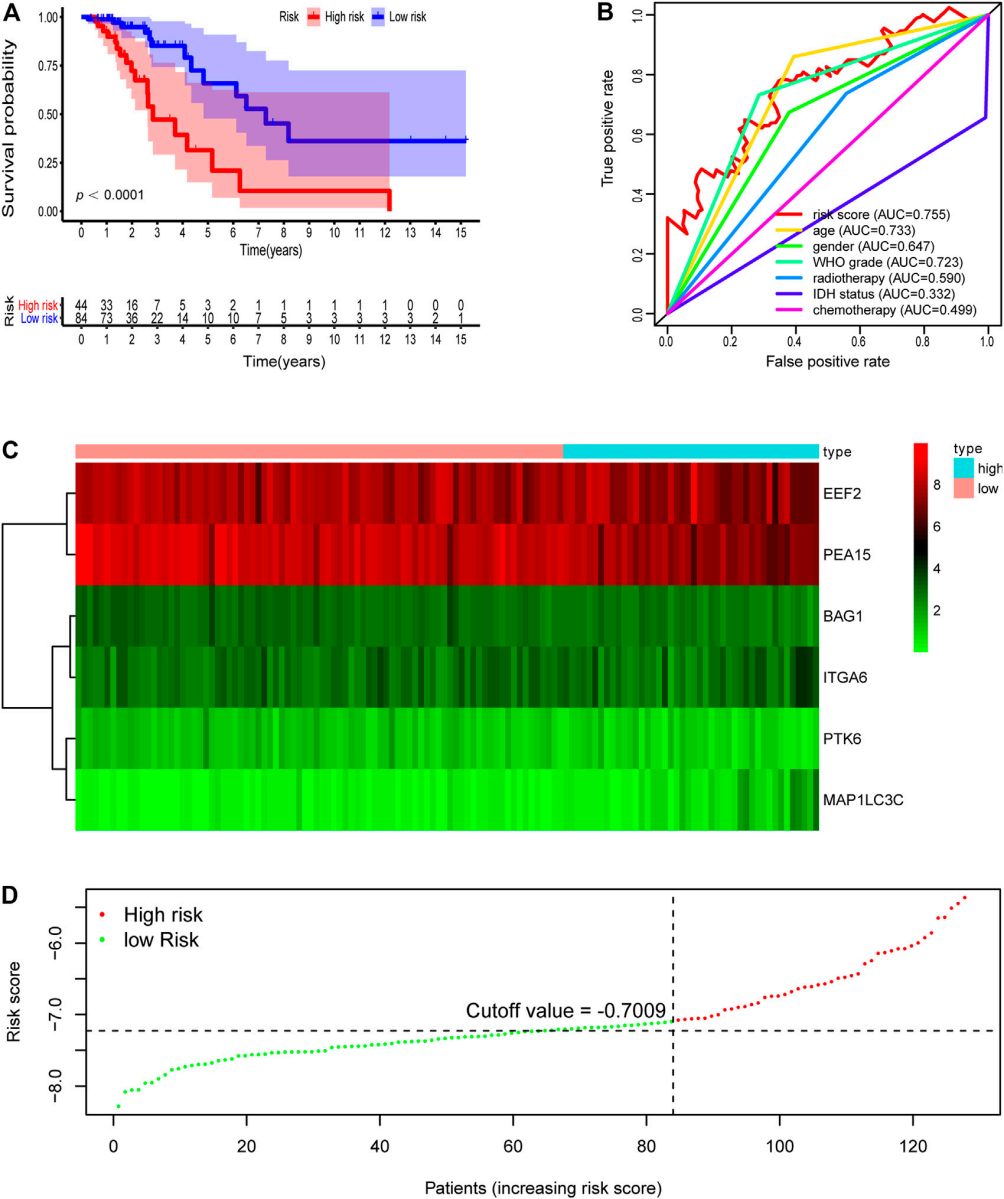
Development of risk score based on the six autophagy-related gene signature of patients with TCGA-LGG validation set. **(A)** Kaplan-Meier plot for overall survival (OS) based on risk score of the six gene based signature of patients with TCGA-LGG validation cohort. **(B)** ROC curve for 5 years OS in validation cohort. **(C)** Heatmap of the six autophagy-related gene expression in the validation cohort. **(D)** Risk plot of each point sorted based on risk score. The black dotted line is the optimal cutoff (−7.009) classifying patients into low risk and high risk groups.

The validation dataset and the whole dataset were used to predict OS and demonstrate the predictive capacity of the risk model. The risk score in each LGG patient from the validation cohort was calculated based on the formula. Then, we divided the validation cohort into a high-risk group (*n* = 44) and a low-risk group (*n* = 84) depending on the optimal risk cutoff value in the training cohort (risk score = −7.009, Figure 3D). Kaplan-Meier analysis indicated that patients in the high-risk group had a poorer prognosis compared to those in the low-risk group (log-rank *p* = 7.382e-05, Figure 3A). The heatmap displayed that six autophagy-related expression profiles between low- and high-risk groups in the validation cohort (Figure 3C). Besides, univariate and multivariate analysis revealed that the risk score was significantly associated with OS after adjustment for other clinical parameters such as age, gender, grade, radiotherapy, chemotherapy, and IDH status (Table 3). Moreover, The ROC curves for 5 years overall survival indicated that the risk score has the best predictive capacity of OS (AUC = 0.755) among the clinical parameters (Figure 3C).

**FIGURE 4:**
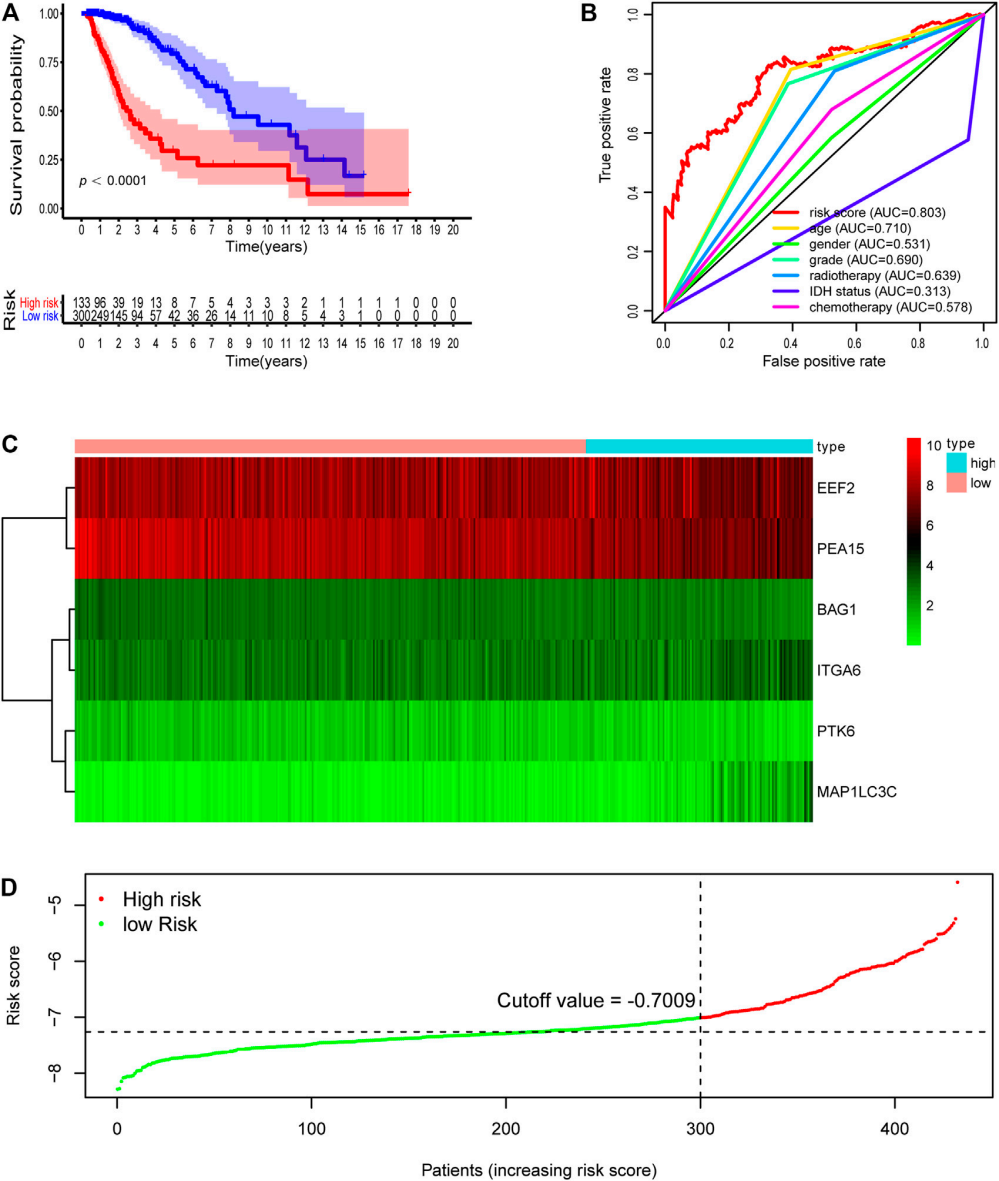
Development of risk score based on the six autophagy-related gene signature of patients with TCGA-LGG whole set. **(A)** Kaplan-Meier plot for overall survival (OS) based on risk score of the six gene based signature of patients with TCGA-LGG whole cohort. **(B)** ROC curve for 5 years OS in whole cohort. **(C)** Heatmap of the six autophagy-related gene expression in the whole cohort. **(D)** Risk plot of each point sorted based on risk score. The black dotted line is the optimal cutoff (−7.009) classifying patients into low risk and high risk groups.

We then further demonstrated the prognostic predictive capacity of the six autophagy-related genes signature in the whole dataset and achieved similar findings. As shown in Figure 4D, the optimal risk cutoff value in the training cohort was adopted to separate the whole dataset into a high-risk group (*n* = 133) and a low-risk group (*n* = 300). KM analysis also revealed that high-risk patients had a poorer prognosis than those in the low-risk group (log-rank *p* value = 0e + 00, Figure 4A). Six autophagy-related expression profiles between low- and high-risk groups in the whole cohort were also showed in a heatmap (Figure 4C). Univariate and multivariate analysis still indicated that the risk signature was significantly associated with overall survival after adjustment for clinical parameters (Table 3). The ROC curves for 5 years overall survival also revealed that the risk score has the best predictive power of OS (AUC = 0.803) than the other traditional clinical parameters (Figure 4B). These results suggested the autophagy-related risk signature performed well in predicting clinical outcomes of LGG patients.

Last, we further compared the predictive capacity of our six autophagy-related genes signature with the two previous models based on autophagy-related genes, by performing ROC curves and Principal component analysis (PCA). The ROC curves for 5 years overall survival revealed that the AUC values of these two published signatures were 0.487 and 0.726 (Supplementary Figure S2), which are lower than our signature. The PCA analysis revealed that our six-autophagy-related genes signature could clearly split the LGG patients into a high- and low-risk group, and presents a best distinction effect compared with other risk models (Supplementary Figure S3). These results indicated that our risk model has greater predictive performance in predicting prognosis compared with other signatures.

### The Association Between the Autophagy-Related Signature and Clinicopathological Factors

**FIGURE 5:**
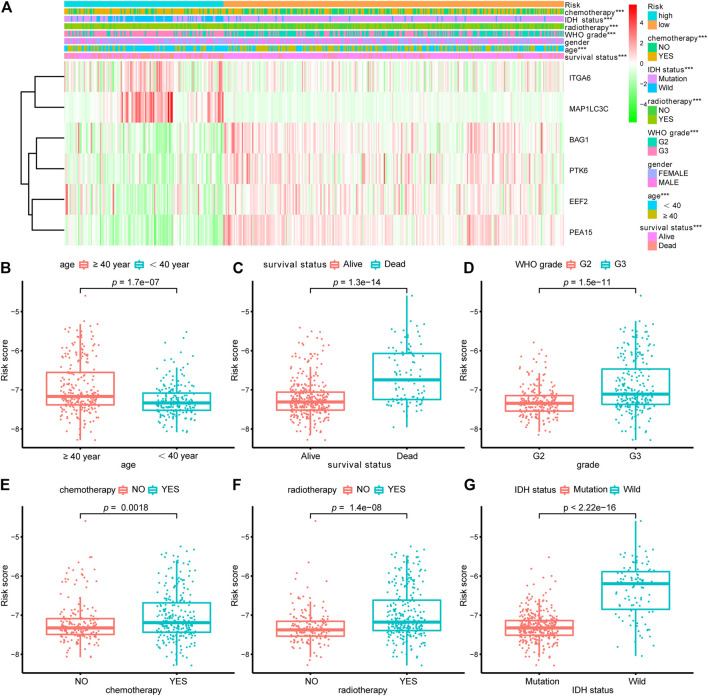
Relationship between the signature risk scores and clinical factors. **(A)** The heatmap showed the relationship between the risk signature and the clinical features (chemotherapy, IDH status, radiotherapy, grade, gender, age and survival status) in the LGG-whole cohort. **(B–G)** The box plots revealed the association between risk score and clinical parameters. *p < 0.01, **p < 0.001, ***p < 0.0001.

To probe the relationship between the risk model and clinical parameters, we firstly used a heatmap to show the distributions of age, gender, WHO grade, radiotherapy, chemotherapy, and IDH status between risk groups in the LGG whole cohort. Figure 5A showed that the risk groups were significantly associated with chemotherapy, radiotherapy, age, WHO grade, and survival status. And there were no significant differences between risk groups for gender. We next assessed the risk scores in various subgroups stratified by age, survival status, grade, chemotherapy, radiotherapy, and IDH status separately. Risk scores in patients above 40 years old were higher than those in the younger age group (Figure 5B). Patients in the alive subtype had obviously lower risk scores than those in the dead subtype (Figure 5C). For the WHO grade, the risk scores in the G3 subgroup were higher than those in the G2 subtype (Figure 5D). The risk scores of patients receiving chemotherapy and radiotherapy were separately higher than those without therapy (Figures 5E,F). With regard to IDH status subtypes, the risk scores significantly increased in the IDH-wild subtype than the IDH-mutation subtype (Figure 5G).

**FIGURE 6:**
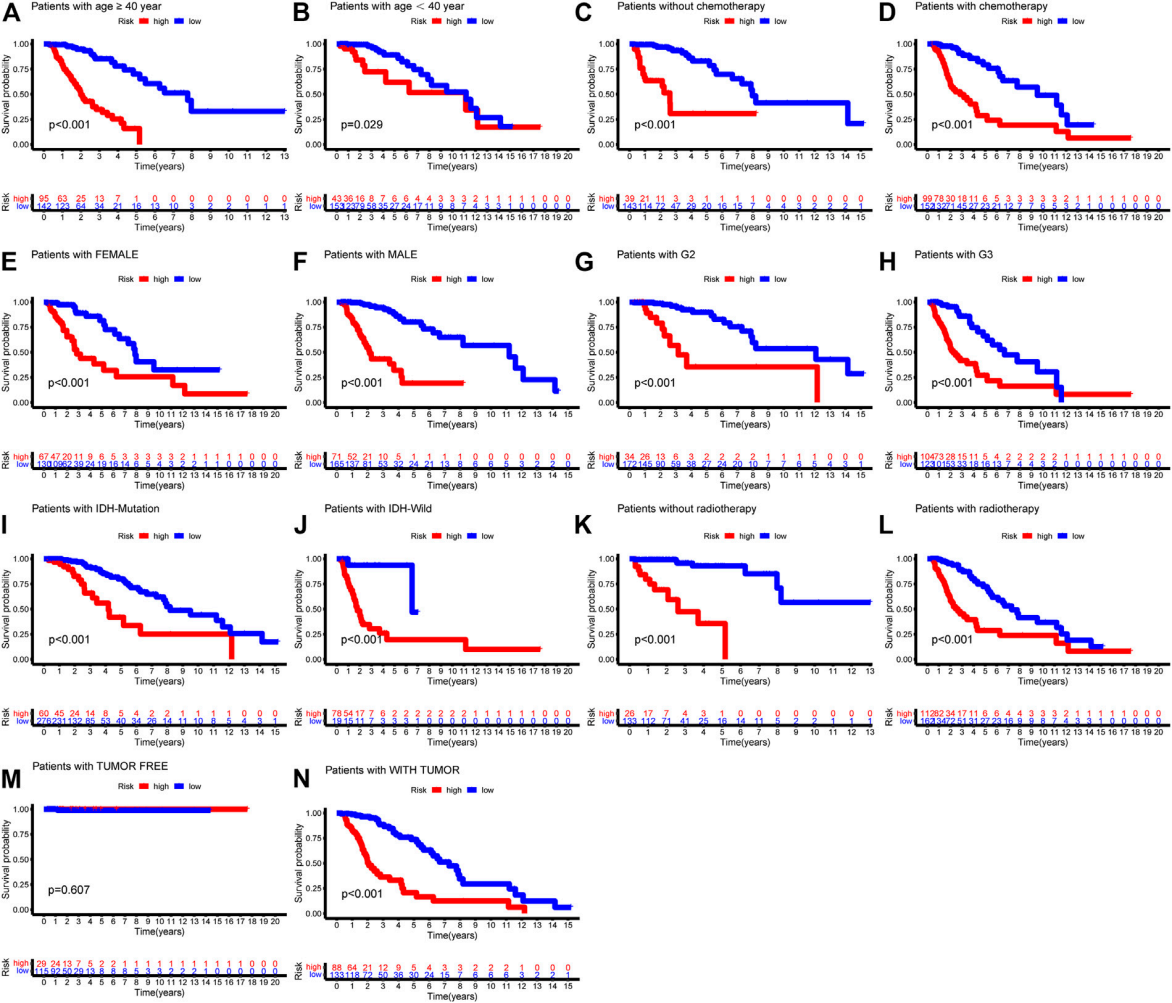
Kaplan-Meier survival curves showed prognostic values of the risk signature in different subgroups of LGG-whole cohort. **(A)** age ≥ 40; **(B)** age < 40; **(C)** without chemotherapy; (**D)** chemotherapy; **(E)** Female; **(F)** Male; **(G)** G2; **(H)** G3; **(I)** IDH-mutation type; (**J)** IDH-wild type; **(K)** without radiotherapy; **(L)** radiotherapy.

We also examined the predictive effects of the six autophagy-related risk model in different subgroups stratified by age, gender, WHO grade, IDH status, and history of radiotherapy or chemotherapy. In the two age subtypes, higher risk scores predicted decreased survival in both age subtypes (Figures 6A,B, *p* < 0.001). Risk scores could separate patients with or without chemotherapy (Figures 6C,D, *p* < 0.001) or radiotherapy (Figure 6K,L, *p* < 0.001) with distinct outcomes. Similar results were also found in the IDH wild- and mutation-type groups (Figures 6I,J, *p* < 0.001), WHO G2 and G3 groups (Figures 6G,H, *p* < 0.0001), and gender groups (Figures 6E,F, *p* < 0.001).

### Establishing a Nomogram as Prognostic Prediction Model

**FIGURE 7:**
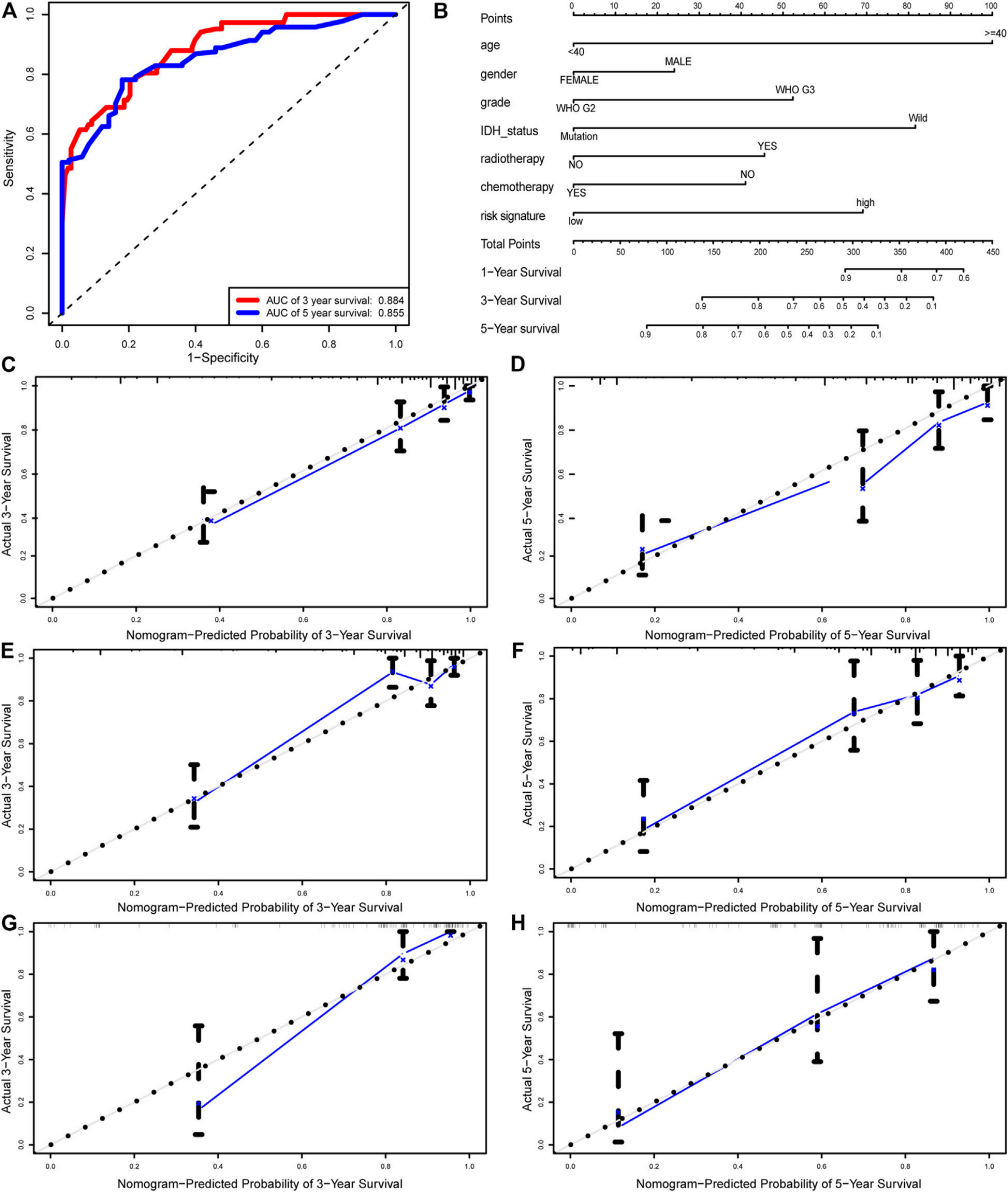
Nomogram built to predict the prognosis of patients with LGG. **(A)** ROC analysis for 3 and 5 years OS predictions with the nomogram. **(B)** A nomogram based on risk score and other clinical parameters for predicting 3 and 5 years OS of LGG. Calibration curves of nomogram for predicting probabilities of 3 years **(C)**, and 5 years **(D)** overall survival of patients in the whole cohort. The calibration plots of for predicting probabilities of 3 years **(E)**, and 5 years **(F)** overall survival of patients in the training cohort. The calibration plots of for predicting probabilities of 3 years **(G)**, and 5 years **(H)** overall survival of patients in the validation cohort. The blue line indicates actual survival.

By integrating the six-autophagy-related signature and six traditional clinical parameters, we constructed a nomogram to predict the survival probability at 3 and 5 years of LGG patients in the whole cohort (Figure 7B). The C-index of the nomogram was 0.845. The AUCs of the nomogram for 3 and 5 years OS predictions were 0.884 and 0.855, respectively (Figure 7A). Meanwhile, the calibration plots also demonstrated a good agreement with predicted and observed values with respect to probabilities of 3 and 5 years survivals (Figures 7C,D). Together, those findings indicated that the nomogram predicts precisely the 3 and 5 years survivals for LGG patients.

### Functional Annotation and Pathway Enrichment Analysis Between the High-Risk Group and Low-Risk Group

**FIGURE 8:**
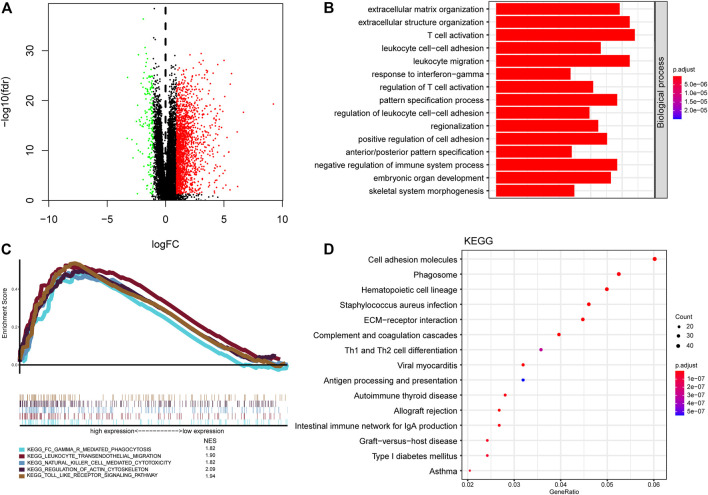
Functional annotation and pathway enrichment analysis of risk groups. **(A)** Volcano plot of differential gene expression analysis between high-risk and low-risk groups. **(B)** Functional annotation for signature using GO biological process. **(C)** Gene set enrichment analysis of curated gene sets obtained from MSigDB Collections. Pathways of interest with significant enrichment in high-risk group was shown. **(D)** Pathway enrichment analysis by KEGG.

To probe the potential biological function of risk groups, both the biological process (BPs) of gene ontology, KEGG, and GSEA were performed. By applying the limma package, the heatmap showed 1904 differentially expressed genes (Figure 8A) between risk groups. Significantly enriched BPs were mainly involved in extracellular matrix organization, T cell activation, and leukocyte cell-cell adhesion (Figure 8B). As for KEGG pathways enriched in these DEGs were cell adhesion molecules, phagosome, Th1 and Th2 cell differentiation, and antigen processing and presentation (Figure 8D). Functional enrichment analysis was then performed between risk groups. GSEA illustrated that the most significant pathways enriched in the high-risk group were Fc gamma receptor-mediated phagocytosis, leukocyte transendothelial migration, natural killer cell mediated cytotoxicity, regulation of actin cytoskeleton, and toll like receptor signaling pathway, while no significant pathways enriched in low-risk group (Figure 8C). A complete list of GSEA results can be found in Supplementary Table S1.

### Differential Expression of Potential Immunotherapy Targets and the Tumor-Infiltrating Immune Cells Between Two Groups

**FIGURE 9:**
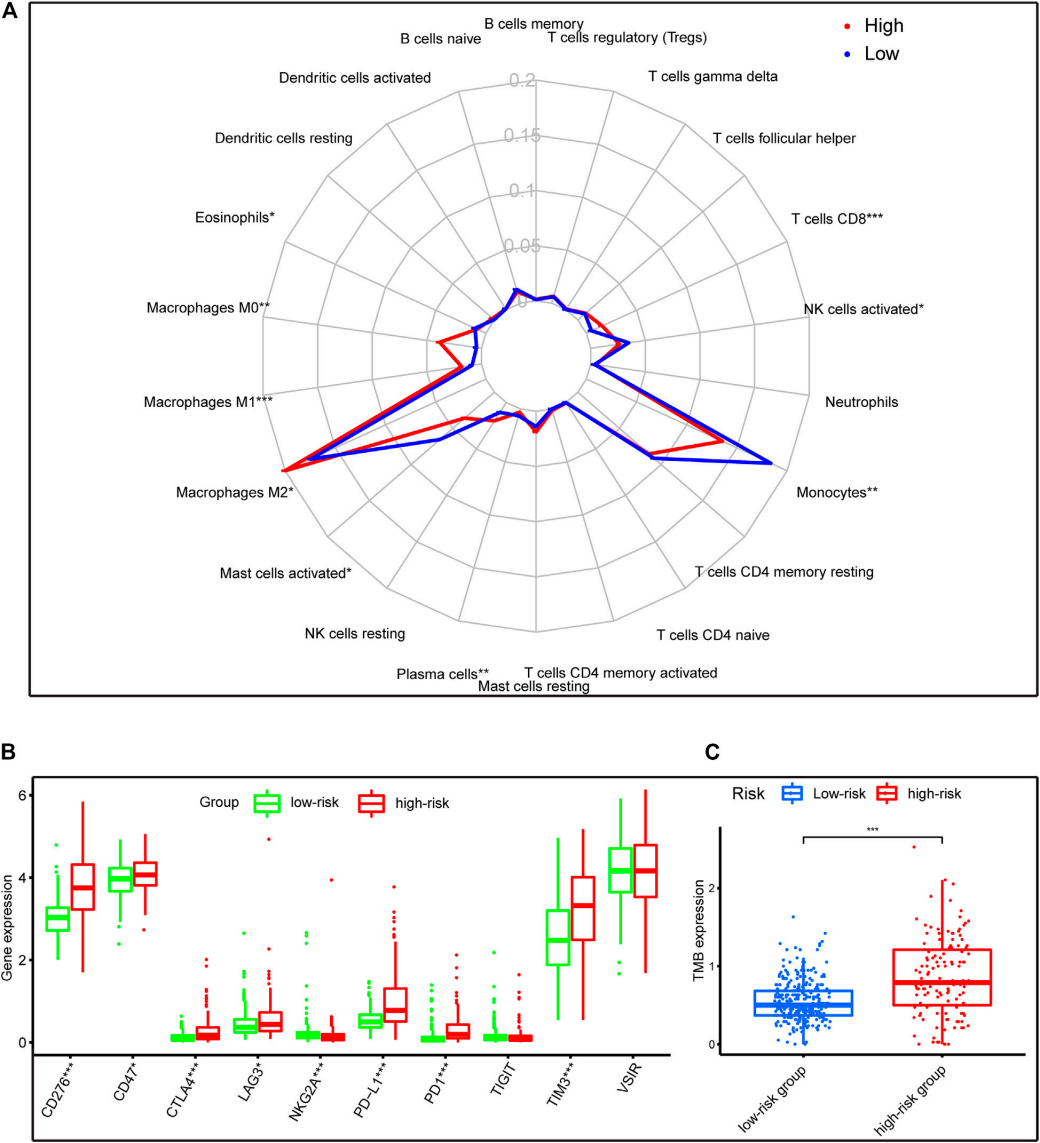
Immune characteristics of risk groups in the whole cohort. **(A)** The radar plot showed the 22 different immune cell levels between high-risk and low-risk groups; **(B,C)** The levels of emerging immunotherapeutic targets and TMB between risk groups. *p < 0.01, **p < 0.001, ***p < 0.0001.

**FIGURE 10:**
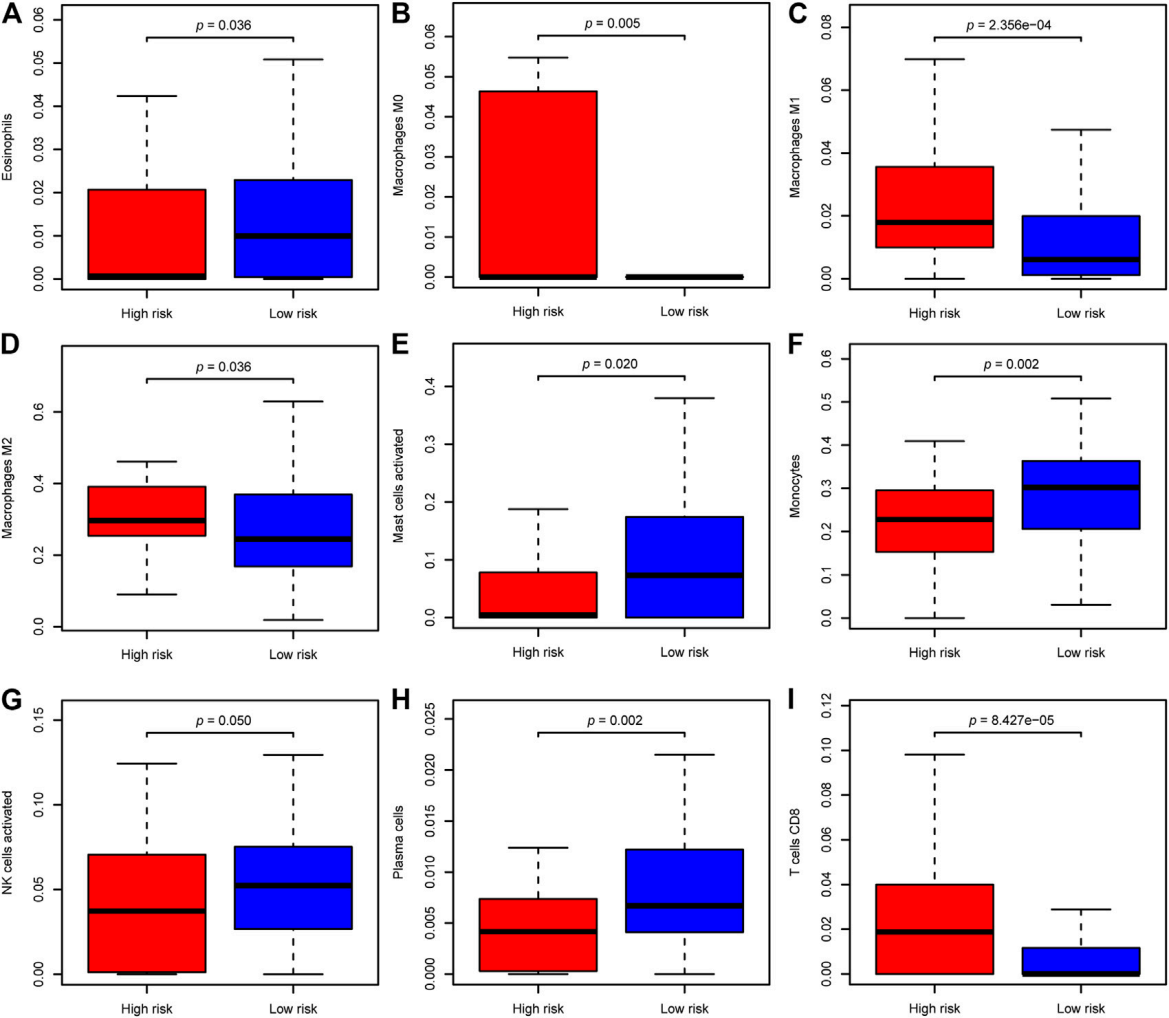
The difference of significantly immune cells between risk groups. **(A)** Eosinophils; **(B)** Macrophages M0; **(C)** Macrophages M1; **(D)** Macrophages M2; **(E)** Mast cells activated; **(F)** Monocytes; **(G)** NK cells activated; **(H)** Plasma cells; **(I)** T cells CD8.

Pathway enrichment between risk groups suggested that autophagy-related genes signature was associated with some immune-related pathways. Thus, we investigated the abundances of the 22 immune cell types for each LGG patient from the whole cohort within the low-risk group and the high-risk group, according to the CIBERSORT algorithm. The comparison of 22 immune cells between risk groups displayed in a radar plot (Figure 9A). Macrophages M0, M1, and M2, and T cells CD8 were obviously increased in the high-risk group than the low-risk group; however, the expression levels of Eosinophils, Mast cells activated, Monocytes, NK cells activated, and Plasma cells were obviously lower in the high-risk group (Figure 10). We also found the gene expressions of multiple promising immunotherapy targets, including CD47, CD276, CTLA-4, LAG3, PD-1/L1, and TIM3, and tumor mutation burden (TMB) were significantly increased in the high-risk group, while the expression levels of NKG2A was significantly upregulated in the low-risk group than in the high-risk group (Figure 9B).

## Discussion

Autophagy has been reported involved in tumor formation and progression, and therapy resistance of multiple cancers, including glioma (Kondo et al., 2005; Mathew et al., 2007; White et al., 2010). Besides, autophagy can alter the tumor or stroma cell immunogenicity within the tumor microenvironment and the response to immunotherapy (Gerada and Ryan, 2020). However, few studies have reported the impact on prognosis and the correlation with immune cells of autophagy in LGG. In this study, the whole samples of the TCGA-LGG project were randomly separated into a training set, and a validation set and the whole set were created for further verification. We established a novel prognosis signature of six autophagy-related genes of LGG in the training dataset, and the signature was verified in the validation and whole datasets. The risk score could well separate patients into a low-risk group and a high-risk group, with a significant difference in overall survival. The AUC of the risk score in predicting the 5 years survival rate in the training set, validation set, and the whole set was 0.837, 0.755, and 0.803, respectively, which suggested that the prognostic signature performed better in predicting clinical outcomes than other traditional clinical factors. The six autophagy-related genes signature could serve as the independent predictive factor of LGG patients, according to multivariate analysis and Kaplan-Meier method. Furthermore, our findings showed that significant differences in tumor immune microenvironment and promising immunotherapy targets between two risk groups in the whole cohort.

Autophagy was involved in a broad range of cellular processes and human diseases, and it is responsible for both carcinogenesis and sensitivity to various therapies in recent years (Mathew et al., 2007; White et al., 2010; Gerada and Ryan, 2020). Hence, it was important to construct the prognostic model based on autophagy-related genes to predict overall survival of LGG patients. Our study first selected 53 DE-ARGs and then identified six genes significantly associated with prognosis. Among them, BAG1, PTK6, EEF2, and PEA15 were protected factors, but ITGA6 and MAP1LC3C were risk factors for LGG patients in univariate Cox regression. BAG1 is a multifunctional protein that associates with multiple cellular processes, such as apoptosis, proliferation, growth, and motility (Ostrom et al., 2013). Besides, BAG1 was reported to be a protective factor in breast cancer (Papadakis et al., 2017). Protein Tyrosine Kinase 6 (PTK6) encodes a cytoplasmic nonreceptor protein kinase, implicated in processes of proliferation, apoptosis, migration, and invasion in cancer cells (Harvey and Crompton, 2004; Shen et al., 2008; Xiang et al., 2008; Harvey et al., 2009; Locatelli et al., 2012; Park et al., 2015). PTK6 was found to be upregulated in many tumor tissues, including breast cancer (Barker et al., 1997), bladder cancer (Xu et al., 2017), non-small cell lung cancer (Zhao et al., 2013), and ovarian cancer (Schmandt et al., 2006), and is associated with adverse outcomes. However, another study showed that PTK6 expression was downregulated in laryngeal squamous cell carcinoma and esophageal squamous cell carcinoma tissues, and low expression levels of PTK6 predicted short survival (Liu et al., 2013; Chen et al., 2014). EEF2 plays an essential role in the translocation of peptidyl-tRNA during protein synthesis. Overexpression of EEF2 was associated with disease progression of lung adenocarcinoma cells (Chen et al., 2011). PEA15 is a 15-kDa phosphoprotein that impedes cell proliferation via inhibiting ERK-dependent proliferation and gene transcription (Formstecher et al., 2001; Bartholomeusz et al., 2006). In addition, PEA15 was found to induce autophagy via activation of the ERK1/2 pathway (Bartholomeusz et al., 2008). ITGA6 is a member of the integrin alpha chain family that conducts signals through interacting with extracellular matrix proteins, serving crucial roles in drug resistance of multiple cancers (Yamakawa et al., 2012; Brooks et al., 2016; Wei et al., 2019). Additionally, overexpression of ITGA6 is associated with shorter overall survival (Zhang et al., 2016; Wei et al., 2019). MAP1LC3A encodes a light chain subunit of the microtubule-associated protein 1-light chain three family, participating in the autophagy and cell mobility process. Giatromanolaki et al. (2014) reported that the overexpression of MAP1LC3A was correlated with impaired autophagic degradation activity, which may facilitate the carcinogenesis of glioblastoma. In addition, another study showed that the MAP1LC3A expression at the surgical margins could be a poor biomarker for clinical prognosis in oral squamous cell carcinoma (Terabe et al., 2018). In summary, BAG1, PTK6, EEF2, PEA15, ITGA6, and MAP1LC3C could serve as predictors for survival in multiple cancers, involving in various biological processes including autophagy. These ATGs may serve as promising prognostic biomarkers and therapeutic targets for guiding LGG therapy.

Then, we established and verified a novel six autophagy-related genes risk model that improves the survival prediction of LGG patients. According to the six autophagy-related signature, LGG patients were separated into a high-risk group and a low-risk group. Patients with high-risk scores predicted worse OS compared to patients with low-risk scores. Afterward, it was successfully validated in the validation and whole datasets, indicating the good reproducibility of this signature. Moreover, Cox regression analysis indicated that the risk score of autophagy-related genes signature is an independent prognostic factor of clinical outcome for LGG patients in multiple cohorts. Additionally, we observed that the risk scores were significantly associated with several clinical factors, including age, grade, IDH mutation status, chemotherapy and radiotherapy. As younger age, low grade glioma and IDH mutation were prognostic factors associated with better outcomes(Taillibert et al., 2004; Cancer Genome Atlas Research et al., 2015; Ostrom et al., 2020), we can speculated that these factors would associated with lower risk scores, which is consistent with our results. Chemotherapy is recommended as an optional treatment alone or in combination with radiotherapy for newly diagnosed LGG patients who cannot undergo gross total resection (Ziu et al., 2015). The higher residual tumor volume (Wijnenga et al., 2018) was reported correlated with shorter OS after adjusting for other clinicopathological factors, suggesting that chemotherapy and radiotherapy might associated with unfavorable outcomes or higher risk scores, which is in accordance with our findings. Moreover, our risk model can classify LGG patients after clinicopathological parameters into high- and low-risk groups with a distinct prognosis, making the risk model can be used to guide individualize treatment. For example, the median age at time of diagnosis for LGG patients around 40 years and the older LGG patients more often associated with unfavorable prognostic factors, including focal deficits, larger residual tumor volumes, compared with younger patients, which may be an explanation for advanced age patients in LGG usually with a poor prognosis (Corell et al., 2018). Additionally, previous study suggested undertreatment of the elderly patients could also contributed to their decreased survival (Kaloshi et al., 2009).Thus, it is crucial to predict the prognosis of the elderly LGG patients, to guide whether the older patients receive the active treatment or not. Fortunately, our autophagy-related genes signature can divide patients with more than 40 years into high- and low-risk groups with distinct outcomes, making the signature can be used to guide individualize treatment. Lastly, we constructed a nomogram comprising the risk score, age, gender, WHO grade, radiotherapy, chemotherapy, and IDH status, Calibration curves of the nomogram predicted the probabilities of 3 and 5 years survival, which corresponded closely with the actual survival rates, suggesting that the nomogram has an excellent predictive performance. Hence, our study identified a nomogram that could help identify LGG patients with a high risk of short survival and guide the selection of better treatment options, which is credible to both physicians and patients. To date, some autophagy-related prognostic classifiers of glioma were published. We further compared the predictive capacity of our risk model with two published signatures (Lin and Lin, 2021; Wang et al., 2021), by performing ROC curves and PCA analysis. These results proved that our six autophagy-related genes signature has the best predictive performance than another signatures, considering different selection criteria of autophagy-related genes yield different outcomes.

The tumor immune microenvironment plays a crucial role in cancer biology (Hanahan and Weinberg, 2011). Previous studies have evaluated the tumor-infiltrating immune cells were deeply involved in glioma development and progression (Perus and Walsh, 2019; Wang et al., 2020). And autophagy and immunity played a momentous role in the tumor microenvironment. Some studies have demonstrated that autophagy plays a critical role in innate immunity as well as the activation of lymphocytes and survival (Germic et al., 2019). Similar to previous findings, our functional analysis also indicated that the significant biological processes and pathways enriched in the high-risk group were involved in some immune-related pathways, such as T cell activation, Th1 and Th2 cell differentiation, and NK cell-mediated cytotoxicity. We further evaluated the relationships of 22 types of immune cell between risk groups in LGG patients. There is a distinctive difference of the cellular component of innate immunity, such as eosinophils, monocytes, macrophages, mast cells, and natural killer (NK) cells between risk groups in the whole cohort. For the eosinophils, the role of autophagy for regulating eosinophil remains largely unknown, for less well studied. Mast cells activated were hypothesized to act as sentinel cells that respond with pathogens and trigger protective immune responses (Piliponsky and Romani, 2018). However, little is known about the mechanism of autophagy for regulating mast cell functions. As for macrophages, the level of macrophages (M0, M1, and M2 macrophages) were significantly increased in the high-risk group than those in the low-risk group, but eosinophils, mast cells activated, monocytes, and NK cells activated were higher in the low-risk group. M2 macrophages comprised the most considerable fraction of macrophages of the high-risk group in our results, which is consistent with the previous study that immunosuppressive M2 macrophages were the dominant type of tumor-associate macrophage (TAM) infiltrations in gliomas (Xu et al., 2020). M2 macrophages contributed to an immunosuppressive tumor microenvironment and promote glioma progression (Xu et al., 2020). Moreover, we found that the high-risk group have a lower abundance levels of NK cell, which have cytotoxic potential against tumor cells and its infiltration is associated with better clinical outcomes (Eckl et al., 2012). In addition, the high-risk group has higher fractions of CD8^+^ T cells. Prior studies have demonstrated that increased CD8^+^ T cells are related to prolonged survival in gliomas (Yang et al., 2010). However, increased expression of immune checkpoints (such as PD-1/L1, LAG3, TIM3) could contribute T cell to a dysfunctional exhausted status following activation (Woo et al., 2012). Our study found that the expression of immune checkpoints was significantly upregulated in the high-risk group compared to the low-risk group. Therefore, the immunosuppressive M2 macrophages, the lower level of NK cells, and the increased expression of immune checkpoints in patients with high risk may be an explanation for their decreased survival.

Cancer immunotherapy is now emerged as the fifth pillar of cancer treatment, with surgery, chemotherapy, targeted pathway inhibition, and radiation (Murciano-Goroff et al., 2020). Immune checkpoint inhibitors (ICIs) have now become the first-line therapies of choice in multiple cancers, such as advanced non-small cell lung cancer and melanoma (Larkin et al., 2015; Reck et al., 2016). However, upregulation of additional immune checkpoints conferring to ICIs resistance, there is a need to identify novel antitumor immune-activating agents. Emerging immunotherapy targets involved in adaptive immunity and innate immune processes, targeting these agents can greatly enhance antitumor immunity, thus eradicating cancer cells (Burugu et al., 2018). For example, LAG-3 has been reported positive expression on the surface of tumor-infiltrating lymphocytes (TILs) of multiple cancers (Deng et al., 2016; Shapiro et al., 2017; Tassi et al., 2017), correlating with aggressive clinical features. In preclinical mouse models, LAG-3 inhibition reenergizes CD8^+^ T cell’s cytotoxicity function and decreases Treg populations, combined with PD-1 inhibitor could improve the antitumor effect (Woo et al., 2012; Huang et al., 2015). Besides, TMB was a potential biomarker for PD-1 inhibitors and patients with high TMB receiving PD-1 inhibition have a higher objective response rate compared to patients with low TMB (Zhu et al., 2019). Our study investigated the immunotherapy target gene expression between different risk groups. The result showed that the gene levels of multiple potential immunotherapy targets, including CD276, CD47, CTLA-4, LAG3, PD-1/L1, and TIM3, and TMB were significantly increased in the high-risk group, while the expression levels of NKG2A was significantly upregulated in the low-risk group than in the high-risk group. Therefore, we speculated that the high-risk patients may benefit from the blockade of these immunotherapy targets in LGG.

The present study has some limitations. Firstly, we built the autophagy-related prognosis signature only with the RNA-seq expression profiles of LGG from TCGA. Although we have separated whole samples into two sets of training cohort and validation cohort, and then verified the performance of the risk signature constructed in the training cohort with the data in the validation and whole cohorts, our prognosis signature would be more powerful with verified in independent external cohorts. Secondly, more details about the molecular mechanisms of six autophagy-related genes and the cross-talk between the autophagy and immune cells in LGG patients required further assessment.

## Conclusion

In summary, we established a reliable autophagy-related six genes signature that can effectively assess the prognosis of LGG patients. Besides, we identified the immune microenvironments and immune targets were different between risk groups, which could be an explanation for poor prognosis in the high-risk group. Furthermore, the six autophagy-related genes risk model might guide the application of immunotherapy in LGG.

## Data Availability

The datasets presented in this study can be found in online repositories. The names of the repository/repositories and accession number(s) can be found in the article/Supplementary Material.
