# Supplementary material for: A Novel Six Autophagy-Related Genes Signature Associated With Outcomes and Immune Microenvironment in Lower-Grade Glioma
Source: Front Genet. 2021 Oct 13;12:698284. doi: 10.3389/fgene.2021.698284 (PMC8548643; doi:10.3389/fgene.2021.698284)

Supplementary Figure 1. The optimal cutoff value of risk score using ROC curve.

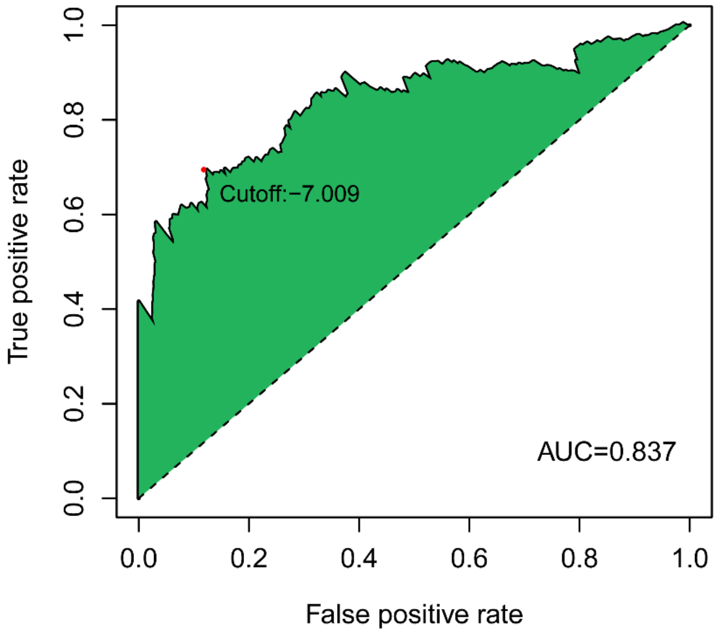

Supplementary Figure 2. The ROC curves for 5-year OS of two published signatures. (A) Wang et al signature (PMID: 32519365) and (B) Lin et al signature (PMID: 33069830).

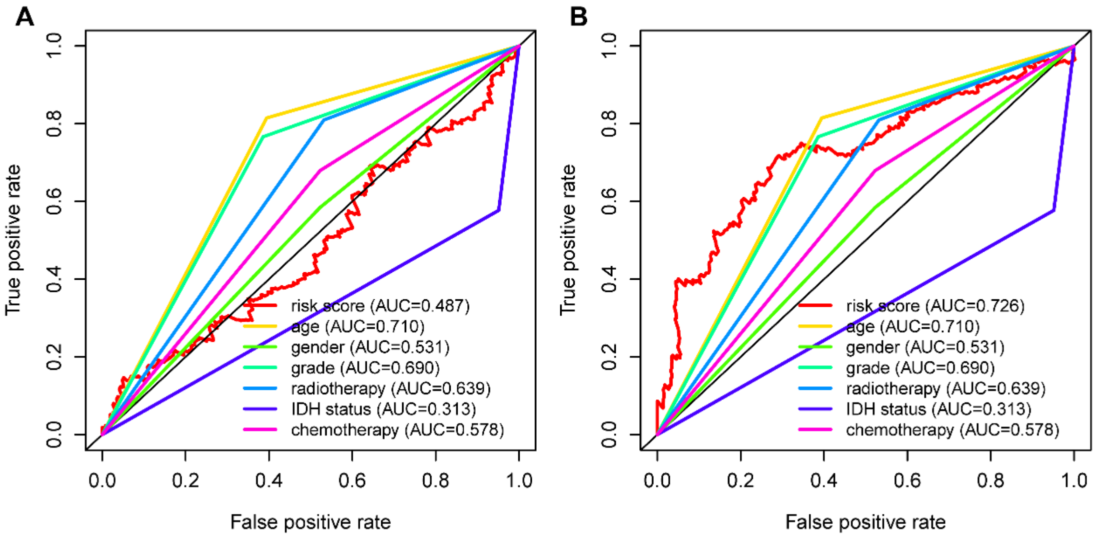

Supplementary Figure 3. PCA among (A) all genes, (B) autophagy-related genes, (C) Wang et al signature (PMID: 32519365), (D) Lin et al signature (PMID: 33069830), and (E) our signature.

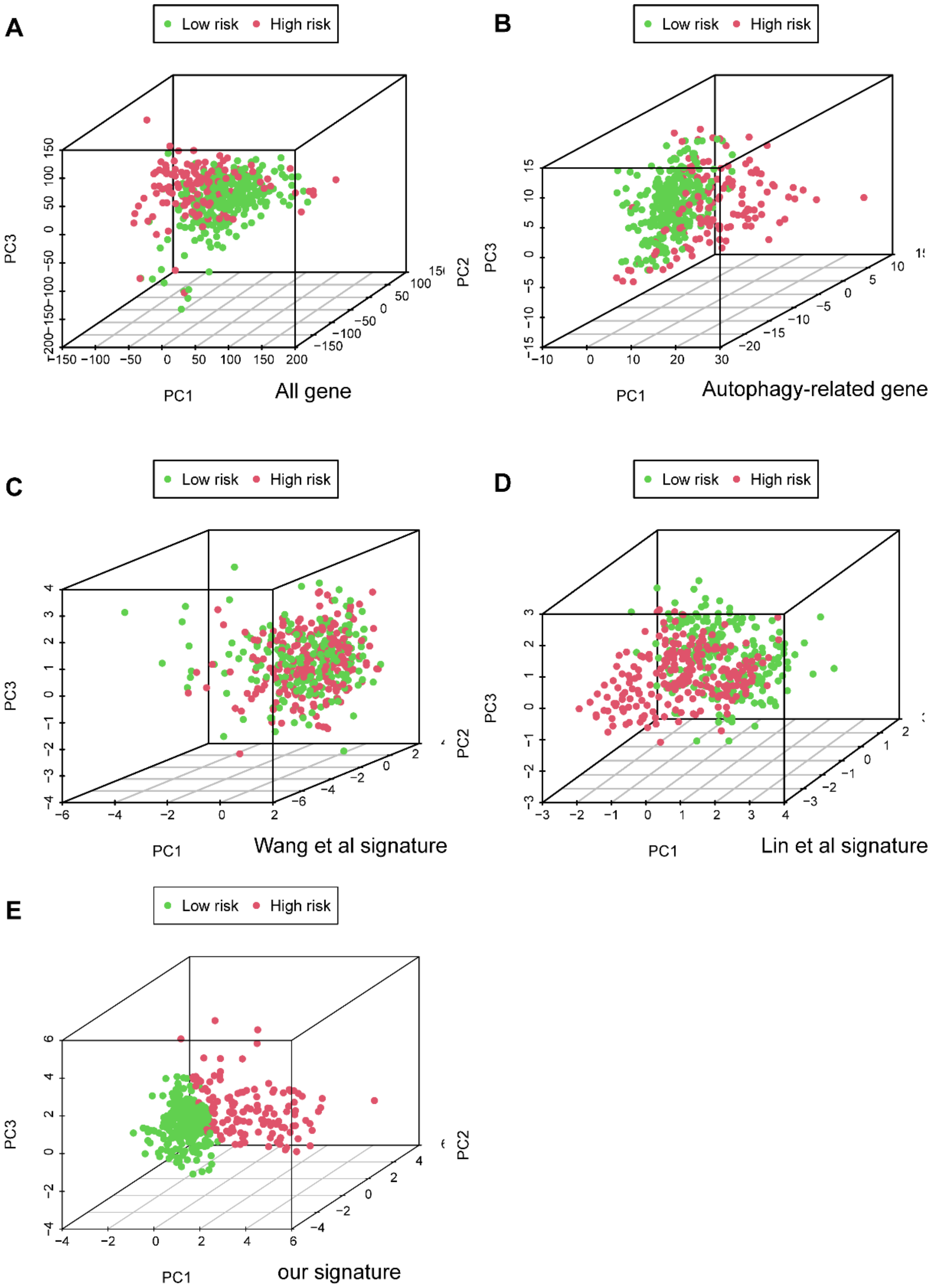

Supplement: Supplementary file 1 [file DataSheet1.pdf]
